# The Molecular Detection and Clinical Significance of *ALK* Rearrangement in Selected Advanced Non-Small Cell Lung Cancer: ALK Expression Provides Insights into ALK Targeted Therapy

**DOI:** 10.1371/journal.pone.0084501

**Published:** 2014-01-03

**Authors:** Ning-Ning Zhang, Yu-Tao Liu, Li Ma, Lin Wang, Xue-Zhi Hao, Zheng Yuan, Dong-Mei Lin, Dan Li, Yu-Jie Zhou, Hua Lin, Xiao-Hong Han, Yan Sun, Yuankai Shi

**Affiliations:** 1 Department of Medical Oncology, Cancer Institute and Hospital, Chinese Academy of Medical Sciences and Peking Union Medical College; Beijing Key Laboratory of Clinical Study on Anticancer Molecular Targeted Drugs, Beijing, China; 2 Department of Pathology, Cancer Institute and Hospital, Chinese Academy of Medical Sciences and Peking Union Medical College, Beijing, China; 3 Department of Medical Record, Cancer Institute and Hospital, Chinese Academy of Medical Sciences and Peking Union Medical College, Beijing, China; Seoul National University, Republic of Korea

## Abstract

**Background:**

This study aimed to elucidate clinical significance of anaplastic lymphoma kinase (*ALK*) rearrangement in selected advanced non-small cell lung cancer (NSCLC), to compare the application of different ALK detection methods, and especially evaluate a possible association between ALK expression and clinical outcomes in crizotinib-treated patients.

**Methods:**

ALK status was assessed by fluorescent *in situ* hybridization (FISH), immunohistochemistry (IHC) and quantitative RT-PCR (qRT-PCR) in 173 selected advanced NSCLC patients. Clinicopathologic data, genotype status and survival outcomes were analyzed. Moreover, the association of ALK expression with clinical outcomes was evaluated in ALK FISH-positive crizotinib-treated patients including two patients with concurrent epidermal growth factor receptor (*EGFR*) mutation.

**Results:**

The positivity detection rate of ALK rearrangement by FISH, IHC and qRT-PCR was 35.5% (59/166), 35.7% (61/171), and 27.9% (34/122), respectively. *ALK* rearrangement was observed predominantly in young patients, never or light smokers, and adenocarcinomas, especially with signet ring cell features and poor differentiation. Median progression-free survival (PFS) of crizotinib-treated patients was 7.6 months. The overall survival (OS) of these patients was longer compared with that of crizotinib-naive or wild-type cohorts, but there was no significant difference in OS compared with patients with *EGFR* mutation. ALK expression did not associate with PFS; but, when ALK expression was analyzed as a dichotomous variable, moderate and strong ALK expression had a decreased risk of death (*P* = 0.026). The two patients with concomitant *EGFR* and *ALK* alterations showed difference in ALK expression, response to EGFR and ALK inhibitors, and overall survival.

**Conclusions:**

Selective enrichment according to clinicopathologic features in NSCLC patients could highly improve the positivity detection rate of *ALK* rearrangement for ALK-targeted therapy. IHC could provide more clues for clinical trial design and therapeutic strategies for ALK-positive NSCLC patients including patients with double genetic aberration of *ALK* and *EGFR*.

## Introduction

Progress in molecular techniques provides better identification and understanding of molecular markers that may have prognostic value and can drive therapeutic decision making for non-small cell lung cancer (NSCLC). In the past decade, a subset of NSCLC patients with epidermal growth factor receptor (*EGFR*) mutation has been attracting much attention because of the high response rates to EGFR tyrosine kinase inhibitors (EGFR-TKIs) [1]. In 2007, a fusion gene of anaplastic lymphoma kinase (*ALK*) with the echinoderm microtubule-associated protein like 4 (*EML4*) in NSCLC was first identified by Soda *et al*. [2], and soon became a novel molecular target for lung cancer treatment. Successful experiences of EGFR targeted therapy have provided a reference model for the fast research progress of *ALK* rearrangement. Crizotinib (ALK/MET/ROS1 inhibitor) was the first clinically available agent that showed remarkable antitumor activity in ALK-positive advanced NSCLC patients. Recently, selection of patients with ALK rearrangement for crizotinib treatment has become a standard in the USA, European Union, China, Japan, and other countries. More importantly, other ALK inhibitors were successively entered into clinical trials [3] and promising to mark a new page of genotype-driven drug development for lung cancer.

The frequency of *ALK* rearrangement ranges from 3% to 7% in unselected NSCLC patients, which could reach to 13% ∼ 18%, if the patient population is selected according to specific clinicopathologic characteristics, especially in young, never-or light smokers with adenocarcinoma [4], [5], [6], [7], [8], [9]. In addition, *ALK* rearrangement was mutually exclusive with *EGFR* and *KRAS* mutations. However, above-mentioned characteristics are not shared by all *ALK* rearrangement carriers. *ALK* fusion has also been found in older patients, smokers [4], patients with *EGFR* mutation [10], [11], [12] and non-adenocarcinoma histological subtypes, such as adenosquamous carcinoma and large cell carcinoma [3], [13]. Therefore, clinicopathologic characteristics are insufficient for screening patients and molecular testing is necessary to determine ALK status [14].

Quantitative real-time polymerase chain reaction (qRT-PCR), immunohistochemistry (IHC) and fluorescence *in situ* hybridization (FISH) are the current methods of choice for ALK testing. However, each method has specific advantages and disadvantages. There is no accepted consensus on which method is preferable. QRT-PCR can detect *ALK* rearrangement at mRNA level and define both *ALK* fusion partner and fusion variant, but it needs high quality of RNA and cannot detect unknown *ALK* rearrangements. In addition, there are a number of *EML4-ALK* variants and non-*EML4-AL*K fusions in NSCLC [15]. Therefore, qRT-PCR is not widely in use in the detection of *ALK* rearrangement. FISH is the current standard method to detect *ALK* rearrangement, since it can detect inversion and translocation irrespective of *EML4-ALK* gene fusion variants and other fusion partners. Importantly, all clinical trials which showed the effectiveness of crizotinib for *ALK*-positive NSCLC patients were based on the Vysis/Abbot ALK break-apart FISH assay. However, FISH is expensive, time consuming and difficult to interpret. So FISH may not be practical for screening every NSCLC patient. IHC is faster, more economical and widely available. Furthermore, IHC with new antibodies and modified protocols has extended its serviceable range for ALK testing. Several published recommendations [16], [17] suggested that ALK FISH analysis can be performed only in IHC-positive cases. However, standard IHC protocols and scoring criteria are lacking, and the correlation between ALK expression and clinical outcomes have not been confirmed to verify the accuracy of IHC.

Therefore, this study collected specimens at the beginning of screening patients for crizotinib clinical trials (PROFILE 1005 or PROFILE 1014) and evaluated ALK status using FISH, IHC and qRT-PCR. Clinicopathologic characteristics and clinical outcomes according to the genotype-specific and therapeutic regimens were analyzed, aiming to elucidate the clinical significance of *ALK* rearrangement in selected advanced NSCLC patients. Furthermore, we compared the application of different ALK detection methods and especially evaluated a possible association between ALK expression and clinical outcomes in ALK FISH-positive crizotinib-treated patients.

## Materials and Methods

### Study Population and Data Collection

Specimens were collected from 173 advanced nonsquamous NSCLC patients who were aiming at undergoing ALK screening for crizotinib clinical trials (PROFILE 1005 or PROFILE 1014) from January 2011 to October 2012. All patients received treatment or consultation from Cancer Institute and Hospital, Chinese Academy of Medical Sciences and Peking Union Medical College and signed informed consent for future molecular analysis. This study was approved by the Institutional Review Boards of the Chinese Academy of Medical Sciences Cancer Institute and Hospital.

Medical records of all patients were reviewed to collect demographic, clinical and pathologic information. Histology was reviewed based on the criteria of the World Health Organization Classification of lung tumors [18] and the IASLC/ATS/ERS multidisciplinary classification of lung adenocarcinoma [19]. We recorded *EGFR* mutation status of patients, which had been determined using a bidirectional sequencing method of *EGFR* exons 18 to 21. We also examined treatment regiments and clinical outcomes. Progression-free survival (PFS) was calculated from the initiation of crizotinib to documented progressive disease (PD) or death from any cause. In order to better elucidate the influences of genotype-specific and therapeutic regimens on patients' overall survival (OS), two types of OS were analyzed. OS1 and OS2 were respectively defined as the time from initial diagnosis of NSCLC and from signed informed consent to death from any cause. OS1 was comprehensive but more influenced by previous treatments. OS2 was more specific to clarify the effects of crizotinib in treating *ALK*-positive advanced NSCLC.

### ALK Test

Specimens were tested by IHC, FISH and qRT-PCR. FISH was conducted with the FDA approved ALK probe kit (Vysis LSI ALK dual-color, break-apart rearrangement probe; Abbott Molecular, Abbott Park, IL) and analyzed according to the kit instructions. ALK IHC was performed according to the protocols provided by the antibody (D5F3, Cell Signaling Technology) manufacturer. Similarly to previous studies [20], [21], IHC results were scored as 0 when no specific staining was apparent within a tumor; 1+, faint staining intensity in more than 10% tumor cells without any background staining; 2+, moderate staining intensity; 3+, strong staining intensity. The cases with sufficient tissues were extracted total RNA from formalin-fixed paraffin-embedded (FFPE) specimens using RNeasy FFPE kit (Qiagen, Germany). For the detection of EML4-ALK, mRNA was first reverse-transcribed into cDNA and qRT-PCR was performed with a commercial EML4-ALK kit (Amoydx, China) including nine known variants (A20, E13; A20, E6a/6b; A20, E20; A20, E15; A20, E14; A20, E18; A20, E2; A20, E17; A20.) on the ABI 7500 Real-Time PCR System (Applied Biosystems, USA).

### Statistical Analysis

Pearson's chi-square test, Fisher's exact test or Kruskal-Wallis test was used for statistical analysis of variables, as appropriate. The Kaplan-Meier approach was used to estimate PFS and OS, and the difference between groups was compared by log-rank test. The hazard ratio (HR) was estimated by proportional hazards regression with a 95% Wald confidence interval (95% CI). Data analysis was done with SAS version 9.2, statistical significance was defined as a two-sided *P*-value<0.05.

## Results

### ALK Detection Results

One hundred and thirty-two resected and forty one biopsied specimens were analyzed both by IHC and FISH; however, no interpretable IHC results were obtained for two patients and no interpretable FISH results for seven patients. QRT-PCR was successfully performed in 122 specimens. ALK alteration was found in 35.5% (59/166), 35.7% (61/171), and 27.9% (34/122) samples by FISH, IHC, and qRT-PCR, respectively. The details of the detection results according to the three methods are summarized in Table 1.

**Table 1:** Distribution of ALK expression grade and ALK variants according to ALK rearrangement in selected advanced NSCLC patients.

*ALK* (FISH)	No.	ALK expression grade (IHC)					*EMl4-ALK* variants^a^ (qRT-PCR)				
		0	1+	2+	3+	N.A.	NO	EA1	EA2	EA3	N.A.
Positive	59	0	5	13	39	2	12	30	1	3	13
Negative	107	103	4	0	0	0	71	0	0	0	36
N.A.	7	7	0	0	0	0	5	0	0	0	2
Total	173	110	9	13	39	2	88	30	1	3	51

Abbreviation: No.: number; N.A.: not available; FISH: fluorescence *in situ* hybridization; IHC: immunohistochemistry; qRT-PCR: quantitative real-time polymerase chain reaction.

^a^ The kit used to detect *EML4-ALK* variants included nine known variants with three primer mixtures; EA1: variant 1 or 3a/3b, EA2: variant 2 or 4/4′, EA3: variant 5′ or 5a/5b.

### Patients' Characteristics and Clinical Outcomes According to Molecular Subtypes

Of the 166 advanced NSCLC patients who were successfully undergone ALK screening by FISH, 59 harbored *ALK* rearrangement including two (3.4%, 2/59) patients with concurrent *EGFR* mutation, 20 showed *EGFR* mutation, 87 were wild type with *ALK*-negative and *EGFR*-negative or *EGFR*-unknown (wild type cohort, WT). The patients most likely to harbor *ALK* rearrangement were young, never or light smokers with poorly differentiated adenocarcinoma, especially with signet ring cell features, comparing with *EGFR* mutation or wild type cohort (Table 2). The two patients with concomitant *ALK* rearrangement and *EGFR* mutation were analyzed separately due to the low incidence rate.

**Table 2:** Characteristics of patients according to molecular status.

Variables	*ALK* Positive	*ALK* Negative (n = 107)		*P* value	
	n = 59 (%)	*EGFR* + n = 20 (%)	Wild-Type ^a^ n = 87 (%)	*ALK* + *vs. EGFR* +	*ALK* + *vs.* Wild-Type
Age at diagnosis, years					
Median	48	58	55	0.009	0.001
Range	(25–74)	(37–72)	(26–77)		
<60	48 (81.4)	12 (60.0)	57 (65.5)	0.103	0.037
≥60	11 (18.6)	8 (40.0)	30 (34.5)		
Sex				0.852	0.264
Male	25 (42.4)	8 (40.0)	45 (51.7)		
Female	34 (57.6)	12 (60.0)	42 (48.3)		
Smoking status ^b^				0.123	0.011
Never/light	52 (88.1)	14 (70.0)	61 (70.1)		
Heavy	7 (11.9)	6 (30.0)	26 (29.9)		
Histopathology ^c^				0.148	0.003
Adeno	48 (81.4)	19 (95.0)	79 (90.8)		
Adeno with SRC features	9 (15.3)	0 (0.0)	1 (1.2)		
Adeno in situ	0 (0)	1 (5.0)	2 (2.3)		
Adenosquamous carcinoma	1 (1.7)	0 (0.0)	4 (4.6)		
Large cell carcinoma	1 (1.7)	0 (0.0)	1 (1.1)		
Differentiation ^d^				0.149	0.003
Poor	31 (52.5)	8 (40.0)	31 (35.6)		
Moderate	7 (11.9)	5 (25.0)	21 (24.1)		
Good	0 (0)	1 (5.0)	8 (9.2)		
Unidentified	21 (35.6)	6 (30.0)	27 (31.0)		

Abbreviation: Adeno, Adenocarcinoma; SRC, signet ring cell.

^a.^ Wild-Type: Patients with *ALK* negative and *EGFR*-negative or *EGF*R-unknown; ^b.^ Heavy smoker indicates a smoker who smoked 10 pack-years or more; ^c.^Fisher's exact test and Adeno with SRC features versus other types; ^d.^ Poor tumor differentiation versus moderate and good tumor differentiation.

**Figure 1:**
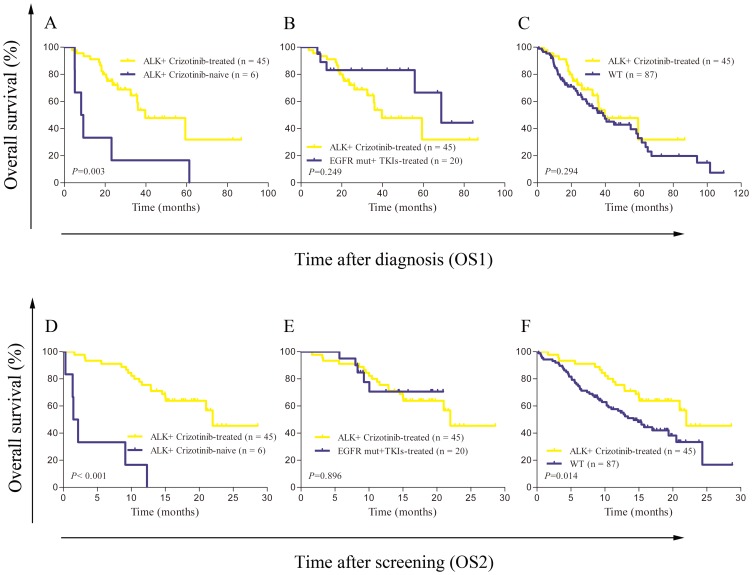
Kaplan-Meier curves of overall survival for advanced NSCLC patients with different genotypes and therapeutic regimens. A, B, C, overall survival calculated from diagnosis (OS1); D, E, F, overall survival calculated from signed informed consent at the screening time (OS2). WT: patients with ALK negative and EGFR-negative or EGFR-unknown; |, indicates censored cases.

In the 59 patients with FISH-positive *ALK* rearrangement, 45 received crizotinib in the phase II clinical trial (PROFILE 1005), 8 were enrolled into phase III clinical trial (PROFILE 1014) and 6 did not participate any clinical trial. Because of the crossover effect of pemetrexed and crizotinib in phase III clinical trial, eight patients were excluded for survival analysis. The other 158 patients were grouped into four types: *ALK* positive with crizotinib-treated (n = 45), *ALK* positive with crizotinib-naive (n = 6), *EGFR* mutation with TKIs-treated (n = 20) and wild type (n = 87). The baseline features, clinical treatments and outcomes are shown in Table 3. *ALK*-positive group showed a dramatically younger age distribution than any other group, received more lines of therapy than *EGFR* mutation group (median, 2.9 *vs.* 1.8; *P* = 0.025) and had more patients received pemetrexed chemotherapy than wild-type group (*P* = 0.020). *ALK*-positive patients with and without crizotinib treatment had statistically significant difference in both OS1 (median, 39.7 *vs.* 8.8 months, *P* = 0.003) and OS2 (median, 22.0 *vs.* 1.8 months, *P*<0.001). There was no significant difference between *ALK*-positive with crizotinib-treated group and *EGFR* mutation with TKIs-treated group both in OS1 and OS2 (*P* = 0.249 and *P* = 0.896, respectively). Additionally, *ALK*-positive with crizotinib-treated patients had longer OS2 than wild-type patients (median, 22.0 *vs.* 14.2 months, *P* = 0.014), but there was no significant difference in OS1 between these two cohorts (39.7 *vs.* 38.7 moths, *P* = 0.294) (Figure 1).

**Table 3:** Characteristics of patients for clinical outcome analysis basing on genotype-specific and therapeutic regimens.

Variables	Group A: *ALK*+ Crizotinib- Treated	Group B: *AL*K+ Crizotinib- Naive	Group C: *EGFR* Mutation with EGFR-TKIs	Group D: Wild-Type ^a^	*P* value		
	(n = 45, %)	(n = 6, %)	(n = 20, %)	(n = 87, %)	A *vs.* B	A *vs.* C	A *vs.* D
Age at diagnosis, years							
Median	47	48	58	55	0.930	0.002	<0.001
Range	(25–73)	(32–60)	(37–72)	(26–77)			
<60	40 (88.9)	5 (83.3)	12 (60.0)	57 (65.5)	0.548	0.019	0.004
≥60	5 (11.1)	1 (16.7)	8 (40.0)	30 (34.5)			
Sex					0.509	1.000	0.201
Male	18 (40.0)	1 (16.7)	8 (40.0)	45 (51.7)			
Female	27 (60.0)	5 (83.3)	12 (60.0)	42 (48.3)			
Smoking status					1.000	0.436	0.132
Never/light	37 (82.2)	5 (83.3)	14 (70.0)	61 (70.1)			
Heavy	8 (17.8)	1 (16.7)	6 (30.0)	26 (29.9)			
Stage at diagnosis					0.886	0.205	0.922
Early	13 (28.9)	1 (16.7)	9 (45.0)	22 (25.3)			
Advanced	32 (71.1)	5 (83.3)	11 (55.0)	52 (59.8)			
Unknown	0 (0)	0 (0)	0 (0)	13 (14.9)			
Stage at screening time					1.000	0.375	0.966
IIIb	5 (11.1)	0 (0)	3 (15.0)	8 (9.2)			
IV	40 (88.9)	6 (100)	17 (85.0)	79 (90.8)			
Brain metastasis					1.000	0.452	0.580
No	32 (71.1)	3 (50.0)	16 (80.0)	62 (71.3)			
Yes	13 (28.9)	2 (33.3)	4 (20.0)	20 (23.0)			
N.A.	0 (0)	1 (16.7)	0 (0)	5 (5.7)			
Bone metastasis					0.776	0.340	0.725
No	26 (57.8)	2 (33.3)	9 (45.0)	50 (57.5)			
Yes	19 (42.2)	3 (50.0)	11 (55.0)	32 (36.8)			
N.A.	0 (0)	1 (16.7)	0 (0)	5 (5.7)			
Prior systemic therapies					0.859	0.025	0.310
Median (range)	2.9 (1–7)	3.0 (1–7)	1.8 (0–6)	2.5 (0–7)			
Pemetrexed at any line					1.000	0.067	0.020
Yes	33 (73.3)	4 (66.7)	10 (50.0)	45 (51.7)			
No	12 (26.7)	2 (33.3)	10 (50.0)	41 (47.1)			
N.A.	0 (0)	0 (0)	0 (0)	1 (1.2)			
TKI at any line					0.627	<0.001	0.499
Ye	21 (46.7)	4 (66.7)	20 (100)	46 (52.9)			
No	24 (53.3)	2 (33.3)	0 (0)	41 (47.1)			
Median OS1 (months)	39.7	8.8	68.8	38.7	0.003	0.249	0.294
Median OS2 (months)	22.0	1.8	unreached	14.2	<0.001	0.896	0.014

Abbreviation: N.A., not available. OS1, overall survival calculated from diagnosis; OS2, overall survival calculated from signed informed consent at the screening time.

^a.^ Wild-Type: Patients with *ALK* negative and *EGFR*-negative or *EGFR*-unknown.

### Survival Analysis of ALK FISH-positive Crizotinib-Treated Patients

**Figure 2:**
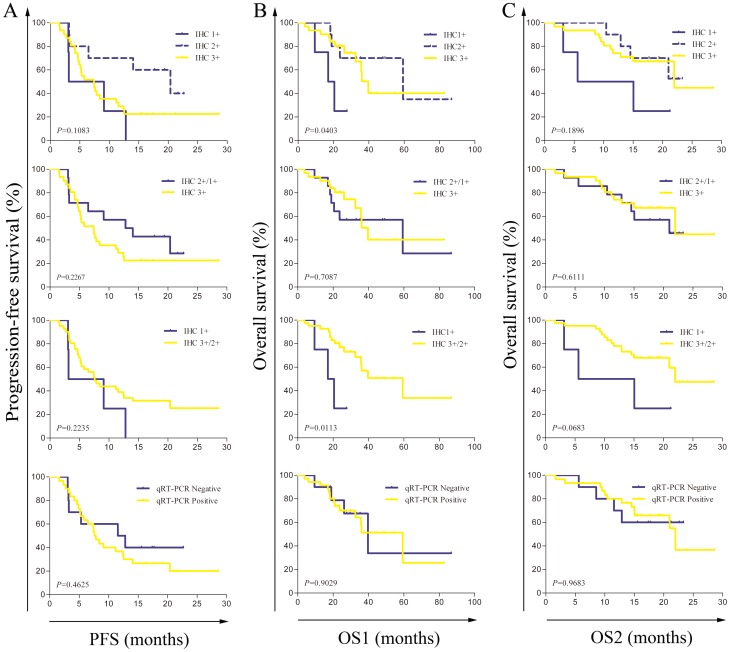
Kaplan-Meier curves of clinical outcomes according to ALK expression and qRT-PCR results for ALK FISH-positive crizotinib-treated patients. A, progression-free survival (PFS); B, overall survival calculated from diagnosis (OS1); C, overall survival calculated from signed informed consent at the screening time (OS2). |, indicates censored cases.

Of the 45 *ALK* FISH-positive crizotinib-treated patients, 2 had no IHC results, 4 stained 1+, 10 stained 2+ and 29 stained 3+. Of these patients, 30 were identified to have *ALK* rearrangement by qRT-PCR, 10 showed negative and 5 had no qRT-PCR results. Up to final follow-up, a total of 33 patients (33/45, 73.3%) presented with PD, and 18 patients (18/45, 40.0%) died. The median PFS of the 45 patients was 7.6 months. We analyzed the potential association between ALK expression and clinical outcomes, such as PFS, OS1 and OS2 using univariate analysis and the results are summarized in Table 4. Multivariate analysis was not performed because of the limitation of the small sample size. Increasing ALK expression, as an ordered variable, did not have any association with PFS (HR = 1.08, 0.60–1.95; *P* = 0.792), OS1 (HR = 0.67, 0.33–1.37; *P* = 0.275) or OS2 (HR = 0.71, 0.36–1.40; *P* = 0.325). However, when ALK expression was taken as a dichotomous variable (i.e., IHC scoring 3+/2+ *vs.* 1+), a IHC score of 3+/2+ significantly predicted improved OS1 (HR = 0.23, 0.06–0.84; *P* = 0.026), but no significant association with OS2 (HR = 0.35, 0.10–1.22; *P* = 0.098) and PFS (HR = 0.49, 0.17–1.42; *P* = 0.187) was observed. When comparing IHC scoring 3+ with 2+/1+, there was no any association with PFS (HR = .52, 0.69–3.32, *P* = 0.30), OS1 (HR = 0.88, 0.34–2.30, *P* = 0.792) and OS2 (HR = 0.84, 0.32–2.17, *P* = 0.713). The Kaplan-Meier curves for PFS, OS1 and OS2 as a function of ALK expression (3+ *vs.* 2+/1+, 3+/2+ *vs.*1+ and 3+ *vs.* 2+ *vs.* 1+, respectively) are shown in Figure 2. In addition, we also analyzed the results of qRT-PCR and found that there was no significant difference between negative and positive patients in PFS, OS1 and OS2 (Figure 2).

**Table 4:** Univariate analysis for progression-free survival and overall survival of biological and clinical parameters for the 45 ALK positive crizotinib-treated patients.

Variables	PFS		OS1		OS2	
	HR (95% CI)	*P* value	HR (95% CI)	*P* value	HR (95% CI)	*P* value
Age at diagnosis (≥60 *vs.*<60 years)	1.21 (0.42–3.46)	0.723	1.25 (0.28–5.61)	0.768	0.96 (0.22–4.18)	0.953
Sex	0.70 (0.35–1.40)	0.316	0.95 (0.37–2.47)	0.920	0.86 (0.33–2.26)	0.757
ECOG (continuous)	2.06 (1.17–3.61)	0.012	2.79 (1.24–6.26)	0.013	1.79 (0.86–3.69)	0.117
Smoking status (heavy *vs.*non or light smokers)	0.91 (0.39–2.32)	0.910	1.87 (0.66–5.29)	0.24	2.17 (0.76–6.18)	0.146
Stage at diagnosis (advanced *vs.* early stage)	1.02 (0.48–2.16)	0.956	2.08 (0.67–6.44)	0.205	1.54 (0.51–4.67)	0.450
Stage at screened time (IV *vs.* IIIb)	1.09 (0.33–3.60)	0.884	24.8 (0.02–26532.93)	0.367	25.1 (0.05–13501.95)	0.315
Brain metastasis	1.37 (0.66–2.83)	0.399	0.59 (0.19–1.80)	0.351	0.75 (0.25–2.30)	0.617
Bone metastasis	1.81 (0.91–3.61)	0.093	1.78 (0.67–4.68)	0.245	1.92 (0.76–4.88)	0.169
Line of therapy (continuous)	0.89 (0.73–1.09)	0.261	0.82 (0.60–1.12)	0.216	0.98 (0.74–1.29)	0.865
Histology (adeno with SRC features *vs.* other types)	0.91 (0.43–2.56)	0.907	0.81 (0.23–2.85)	0.743	0.82 (0.25–3.00)	0.821
Type of therapy (pemetrexed therapy or not)	0.80 (0.38–1.67)	0.546	0.88 (0.28–2.75)	0.831	1.21 (0.39–3.74)	0.737
Type of therapy (EGFR-TKIs therapy or not)	1.08 (0.54–2.15)	0.828	1.37 (0.49–3.84)	0.551	2.01 (0.74–5.49)	0.171
Type of therapy (radiation therapy or not)	1.30 (0.65–2.61)	0.457	0.77 (0.29–2.05)	0.596	0.95 (0.36–2.55)	0.919
IHC scoring (continuous)	1.08 (0.60–1.95)	0.792	0.67 (0.33–1.37)	0.275	0.71 (0.36–1.40)	0.325
IHC scoring (dichotomous) (IHC2+/3+ *vs.* 1+)	0.49 (0.17–1.42)	0.187	0.23 (0.06–0.84)	0.026	0.35 (0.10–1.22)	0.098
IHC scoring (dichotomous) (IHC3+ *vs.* 2+/1+)	1.52 (0.69–3.32)	0.300	0.88 (0.34–2.30)	0.792	0.84 (0.32–2.17)	0.713

Abbreviation: PFS, progression-free survival; HR, hazard ratio; CI, confidence interval; Adeno: adenocarcinoma; SRC: signet ring cell. IHC: immunohistochemistry; ECOG, Eastern Cooperative Oncology Group; EGFR-TKI, epidermal growth factor receptor tyrosine kinase inhibitor; OS1, overall survival calculated from diagnosis; OS2, overall survival calculated from signed informed consent at the screening time.

In the univariate analysis of clinical factors, patients' age, sex, smoking status, stages at diagnosis and screening time, brain and bone metastasis at screening time, previous lines of therapy, histology, and type of therapy (including pemetrexed, EGFR-TKIs and radiation therapy) were not associated with clinical outcomes. Eastern Cooperative Oncology Group (ECOG) performance status showed prognostic significance for PFS (HR = 2.06, 1.17–3.61; *P* = 0.012), OS1 (HR = 2.79, 1.24–6.26; *P* = 0.013), but no prognostic significance for OS2 (HR = 1.79, 0.86–3.69; *P* = 0.117).

### Profiles of Patients with Concomitant ALK Rearrangement and EGFR Mutation

**Figure 3:**
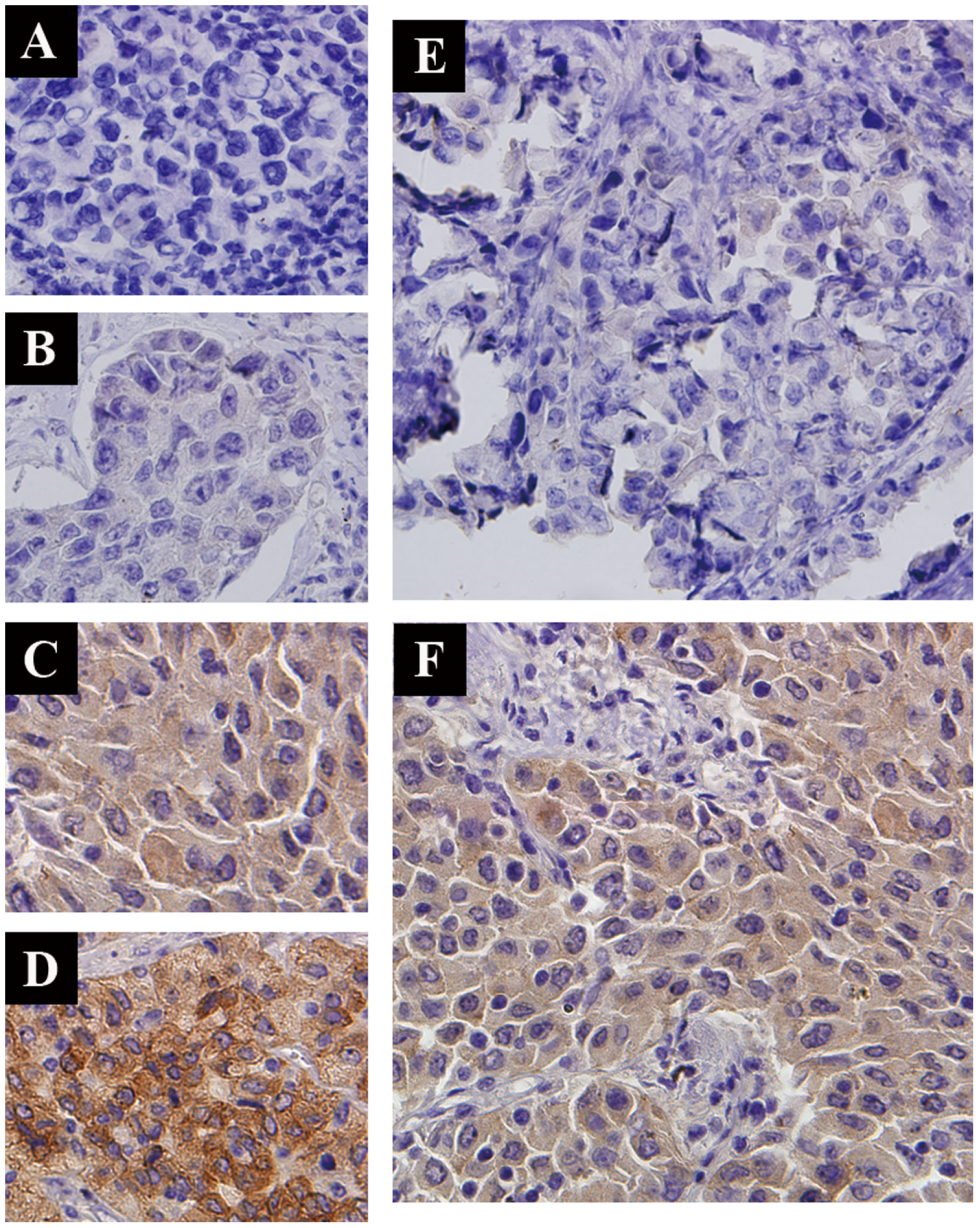
ALK immunohistochemical staining. A, no staining (score, 0); B, faint staining (score, 1+); C, moderate staining (score, 2+); D, strong staining (score, 3+); E, ALK protein staining of patient with concurrent ALK and EGFR mutation (patient 1, score, 1+); F, ALK protein staining of patient with concurrent ALK and EGFR mutation (patient 2, score, 2+). Magnification, 400×.

During our data collection, two patients harbored coexisting *ALK* rearrangement and *EGFR* mutation (one had exon 19 deletion and the other had exon 21 mutation). They shared some clinicopathologic features, including male sex, young age (42 years and 46 years, respectively), adenocarcinoma and heavy smoking status. Patient 1 was in stage IV and patient 2 was in stage IIIA at diagnosis, and both of them were in stage IV at screening time. In addition, patient 1 was judged inoperable and patient 2 was underwent surgery. Patient 1 received EGFR-TKI (erlotinib) as first-line therapy and the best response was partial remission (PR). Patient 2 received EGFR-TKI (erlotinib) as fourth-line therapy and the best response was stable disease (SD). PFS of erlotinib was 6.2 months (patient 1) and 3.6 months (patient 2). FISH and IHC for ALK status were performed in both patients, and qRT-PCR was just performed in patient 2. Both patients were ALK FISH positive, but patient 1 showed faint ALK expression. Patient 2 had moderate ALK expression (Figure 3) and showed positive by qRT-PCR. Both of them were enrolled into the phase II clinical trial of crizotinib, and PFS of crizotinib was 3.1 months (patient 1) and 21.7 months (patient 2). The OS1 of patient 1 and patient 2 was 20.5 and 48.0 months, respectively. And patient 2 did not present with PD during crizotinib treatment and was still alive at the final follow-up.

## Discussion

*ALK* rearrangement has become a clinically important marker in selecting advanced NSCLC patients for molecular targeted therapy. However, the incidence of *ALK* rearrangement is relatively low, even in the previously reported enriched population. It is difficult to identify the subsets of *ALK*-positive patients. Therefore, enriching patients with greatest degree and accurate determination of *ALK* rearrangements are the key importance to screen appropriate candidates for ALK inhibitors. Due to the disadvantages of the current standard FISH, previous comparative studies suggested that IHC, compared with qRT-PCR, was a promising prescreening method for patients with *ALK* rearrangement. But clinical outcomes data were lacking to understand comprehensively which testing platform was the most accurate to predict response to targeted therapy. Our data may offer new insights to the molecular detection in identifying appropriate individuals for ALK inhibitors treatment.

*ALK* is one of the newest tyrosine-kinase targets in NSCLC. It is aberrantly activated due to a chromosomal rearrangement, leading to the expression of an oncogenic fusion kinase, ALK protein. The most common rearrangement subtype is *EML4-ALK* [15]. In NSCLC, *ALK* rearrangement is associated with distinct clinicopathologic features, including young age at onset and adenocarcinoma histology in patients with a history of never or light smoking. Generally, the clinicopathologic features of *ALK*-positive patients in our study were similar to previous studies reported [5], [22]. Notably, *ALK* rearrangement was more common in poor tumor differentiation in our selected population. To date, only Takahashi *et al.* [23] reported that *ALK* rearrangement was associated with poor differentiation status in five *ALK*-positive patients, our data further affirmed this finding in a relatively large sample size.

Approximately one third of patients harbored *ALK* rearrangement in our study, which was higher than previously reported range. All recruited patients were suggested by the oncologists to screen for ALK targeted therapy based on clinical considerations (adenocarcinoma, advanced stage, young age, non- or light smoking, or previously treated with cytotoxic regimens and/or targeted therapy). The majority of these patients shared some above-mentioned features, and this may actually affect the frequency of positive detection. *ALK* rearrangement presented a relatively rare incidence rate in unselected population. These data indirectly indicated that the detection rate of *ALK* gene translocation could be highly improved based on the selective enrichment according to clinicopathologic features. This information could be helpful for screening *ALK* positive patients with the greatest degree in clinical routine practice.

In absence of ALK targeted agents, the prognostic value of *ALK* alterations is controversial [24], [25], [26]. However, with the development of molecular targeted agents, such as crizotinib, positive ALK status was a positive predictive marker for ALK inhibitor therapy [27]. We analyzed survival according to molecular status and therapy regimens, and found that crizotinib prolonged OS of *ALK*-positive patients. Crizotinib-naive patients showed a generally poor outcome, worse than that of the general population of NSCLC patients. Thus, *ALK* rearrangement is not a favorable prognostic factor in advanced NSCLC. Of note, these results should be interpreted with caution because crizotinib-naive patients with small sample size had worse performance status than that of patients recruited into crizotinib clinical trials. In addition, *ALK*-positive crizotinib-treated patients had no difference in OS, compared with EGFR mutation TKIs-treated patients, although their survival was numerically shorter. This may be due to the difference in the number of prior lines of therapy between the two groups. *EGFR*-positive patients represent a well established, TKI-sensitive paradigm. Most *EGFR*-positive patients immediately received EGFR-TKIs treatment after molecular diagnosis and got superior survival. Moreover, crizotinib prolonged *ALK*-positive patients' OS calculated from screening time, but did not prolong OS calculated from diagnosis compared with wild-type patients. It was suggested that *ALK*-positive NSCLC patients might be particularly responsive to pemetrexed chemotherapy [28], [29]. Therefore, small events within *ALK*-positive crizotinib-treated group, patients with unknown *EGFR* status in wild-type group and different treatment history of pemetrexed between the two groups might have confounded the results. Although our study had the limitations of a single-center, restricted statistical power because of the small sample size, and confounding factors, including performance status, prior treatment history, unknown *EGFR* status and small events, we still could clearly reveal the effects of crizotinib in treating *ALK*-positive advanced NSCLC.

FISH is currently used standard based on the pivotal studies of crizotinib. However, some shortcomings of the ALK FISH assay have been reported. The clinical trials of crizotinib recruited patients who were positive by FISH only, but it was later noted [30] that patients with double-positive of FISH and IHC results had a higher response rate. It was also reported [31] that a patient with ALK IHC-positive and ALK FISH-negative had dramatic response to crizotinib. These findings suggested that there might have been patients with false-positive and -negative results by FISH. In addition, the uninformative rate of FISH among tumors reported as IHC-positive was high [32]. Recently, several published recommendations [16], [17] suggested that FISH analysis could be performed only in IHC-positive cases and other recommendation [33] indicated that IHC, if carefully validated, may be considered as a screening methodology to select specimens for FISH testing; qRT-PCR was not recommended as an alternative to FISH. But to date, previous studies have not compared the prognostic power of IHC or qRT-PCR. In theory, each biologically relevant *ALK* rearrangement leads to overexpression of ALK protein, which is the true drug target. In our study, patients with faint staining intensity had inferior clinical outcomes and poor response to ALK inhibitor. Although multivariate analysis could not be performed due to the small sample size of crizotinib-treated patients, we did demonstrate by univariate analysis that tumor with moderate and strong ALK expression marginally predicted improved OS2 and significantly predicted a decreased risk of death. These findings were unequivocally substantiated by the Kaplan-Meier survival curves, which demonstrated a significantly superior overall survival of patients with moderate or strong ALK expression. The positive association of ALK expression with improved survival is intriguing and surprising. Moreover, ALK positive and negative patients classified by qRT-PCR in crizotinib-treated patients had no significant difference in clinical outcomes. To our knowledge, this is the first report which elucidated the association of ALK expression and qRT-PCR with clinical outcomes to evaluate the application of ALK detection methods. Considering the affordability and sensitivity of IHC and qRT-PCR, an IHC based ALK test may represent a reliable and cost-effective screening strategy in identifying patients who might benefit from ALK inhibitors. Due to the limitation of small sample size, our data support the need for large-scale and prospective biomarker studies to validate this diagnostic strategy for ALK-positive NSCLC.

*ALK* rearrangement and *EGFR* mutation are generally mutually exclusive [34]. But the coexistence of *ALK* and *EGFR* has been successively reported (>1% of all treated NSCLC) [35], [36], [37]. In our study, the incidence rate was 3.4%. However, Wang *et al.* [11] reported a higher frequency (15%) of this double genetic aberration in Chinese patients. It suggested that *EGFR* mutation and *ALK* rearrangement may arise independently during oncogenesis and may act synergistically. The two patients harboring the double genetic aberrations shared some similar characteristics, but there was obvious difference in ALK expression and response to EGFR and ALK inhibitors. Patient 2 with moderate ALK expression had better response to crizotinib, but inferior response to erlotinib than the patient with faint ALK expression. It was reported [5] that ALK fusion was strongly associated with resistance to EGFR-TKIs. A patient with concomitant *EGFR* mutation and *ALK* rearrangement, demonstrated no ALK expression by IHC with an *ALK* rearrangement featured by an isolated 3′ FISH signal, and presented the most durable response to an EGFR-TKI [37]. While, another concurrent case of *ALK* rearrangement and *EGFR* mutation, presented ALK expression, but EGFR-TKI was not effective [36]. Until now, the most effective treatment for patients with advanced NSCLC harboring the double mutation is still an open question. However, our data provided a clue for the treatment of patients with the coexistence of *EGFR* mutation and *ALK* rearrangement by evaluating ALK expression, but further research is needed to confirm the appropriate treatment for these patients.

In summary, in the era of ALK-targeted inhibitors, selective enrichment according to clinicopathologic features in NSCLC patients could efficiently screen ALK positive candidates for molecular targeted therapy. IHC could be a pre-screening and supplementary algorithm to provide more clues for clinical trial design and therapeutic strategies for NSCLC patients harboring *ALK* rearrangement, including patients with double genetic aberration of *ALK* and *EGFR*. However, the true prognostic value of ALK expression should be further validated in future prospective studies.
